# Phenotypic miRNA Screen Identifies miR-26b to Promote the Growth and Survival of Endothelial Cells

**DOI:** 10.1016/j.omtn.2018.08.006

**Published:** 2018-08-18

**Authors:** Andrea Martello, David Mellis, Marco Meloni, Alison Howarth, Daniel Ebner, Andrea Caporali, Ayman Al Haj Zen

**Affiliations:** 1 British Heart Foundation Centre for Cardiovascular Science, QMRI, University of Edinburgh, Edinburgh EH16 4TJ, UK; 2 Target Discovery Institute, Nuffield Department of Medicine, University of Oxford, Oxford OX3 7FZ, UK; 3 British Heart Foundation Centre of Research Excellence, Division of Cardiovascular Medicine, Radcliffe Department of Medicine, University of Oxford, Oxford OX3 9DU, UK

**Keywords:** high content screen, miRNA mimic library, miR-26b, endothelial cells, cell growth

## Abstract

Endothelial cell (EC) proliferation is a crucial event in physiological and pathological angiogenesis. MicroRNAs (miRNAs) have emerged as important modulators of the angiogenic switch. Here we conducted high-content screening of a human miRNA mimic library to identify novel regulators of EC growth systematically. Several miRNAs were nominated that enhanced or inhibited EC growth. Of these, we focused on miR-26b, which is a conserved candidate and expressed in multiple human EC types. miR-26b overexpression enhanced EC proliferation, migration, and tube formation, while inhibition of miR-26b suppressed the proliferative and angiogenic capacity of ECs. A combinatory functional small interfering RNA (siRNA) screening of 48 predicted gene targets revealed that miR-26b enhanced EC growth and survival through inhibiting PTEN expression. Local administration of miR-26b mimics promoted the growth of new microvessels in the Matrigel plug model. In the mouse model of hindlimb ischemia, miR-26b was found to be downregulated in endothelium in the first week following ischemia, and local overexpression of miR-26b improved the survival of capillaries and muscle fibers in ischemic muscles. Our findings suggest that miR-26b enhances EC proliferation, survival, and angiogenesis. miR-26b is a potential target for developing novel pro-angiogenic therapeutics in ischemic disease.

## Introduction

Blood vessels remain mostly quiescent throughout adult life. However, in response to injury or pathological conditions, they maintain the capacity to rapidly form a new vascular network from pre-existing vessels in a complex process called angiogenesis.^1^ During sprouting angiogenesis, while tip cells are highly migratory endothelial cells (ECs) that guide the new sprout toward pro-angiogenic gradients, neighboring stalk cells elongate the new sprout by their highly proliferative capacity.^2^ EC proliferation is also involved in other types of post-natal angiogenesis, such as enlargement of pre-existing capillaries and bridging or intussusception of enlarged vessels to form smaller daughter vessels.^3^

Both the survival and growth of the pre-existing capillary network have been demonstrated to be major determinant factors for the formation of a functional vascular network and re-establishing the tissue reperfusion in response to ischemic injury.^4^, ^5^ For instance, vascular endothelial growth factor (VEGF) and other vascular growth factors, such as fibroblast growth factor (FGF), bind to their receptors on ECs and stimulate the downstream PI3K-AKT1-mTOR pathway, which is essential for EC proliferation and survival.^6^, ^7^, ^8^, ^9^, ^10^ Impairment of EC growth and survival pathways causes a deficiency in post-ischemic angiogenesis.^11^, ^12^ Patients with limb ischemia have a lower capillary density of skeletal muscles, and that is related to the functional impairment capacity,^13^ indicating the presence of an insufficient adaptive mechanism of angiogenesis to compensate the lack of blood supply in ischemic muscles. Thereby, the induction of angiogenesis in ischemic vascular disease would be beneficial, including enhancing EC growth, which is a hallmark of angiogenesis.

MicroRNAs (miRNAs) are single-stranded RNAs that target mRNAs with complementary sequences, leading to their transcript destabilization, translational inhibition, or both.^14^, ^15^ Previous reports have demonstrated that the miRNA pathway can be critical for vascular development, post-natal angiogenesis, and pathological angiogenesis.^16^, ^17^, ^18^ Many miRNAs were identified to be essential for angiogenesis and vascular response after injury. For example, it has been reported that miR-221 is required for endothelial tip cell behavior during vascular development.^19^ EC-selective miR-15a transgenic overexpression leads to reduced blood vessel formation and local blood flow perfusion in mouse hindlimbs.^20^ miR-503 caused EC dysfunction and impaired post-ischemic vascular repair.^21^, ^22^ Furthermore, miRNA expression changes have been associated with several vascular diseases.^23^, ^24^, ^25^

Phenotypic high-content screening offers a systematic approach exploring a large number of genome-wide libraries of small interfering RNAs (siRNAs) and miRNAs, allowing the unbiased analysis of a high number of cells, at the single-cell level.^26^, ^27^ The use of this approach has been applied successfully for the identification of miRNAs regulating cellular processes involved in cardiovascular physiology and pathology.^28^, ^29^, ^30^ Here we conducted a comprehensive functional high-content miRNA screening to identify miRNAs triggering EC growth, using an entire human miRNA mimic library, which could be potentially novel targets for therapeutic angiogenesis. We identified miR-26b as an enhancer of EC survival and growth *in vitro* and *in vivo*.

## Results

### Screening for miRNAs Regulating EC Growth

**Figure 1:**
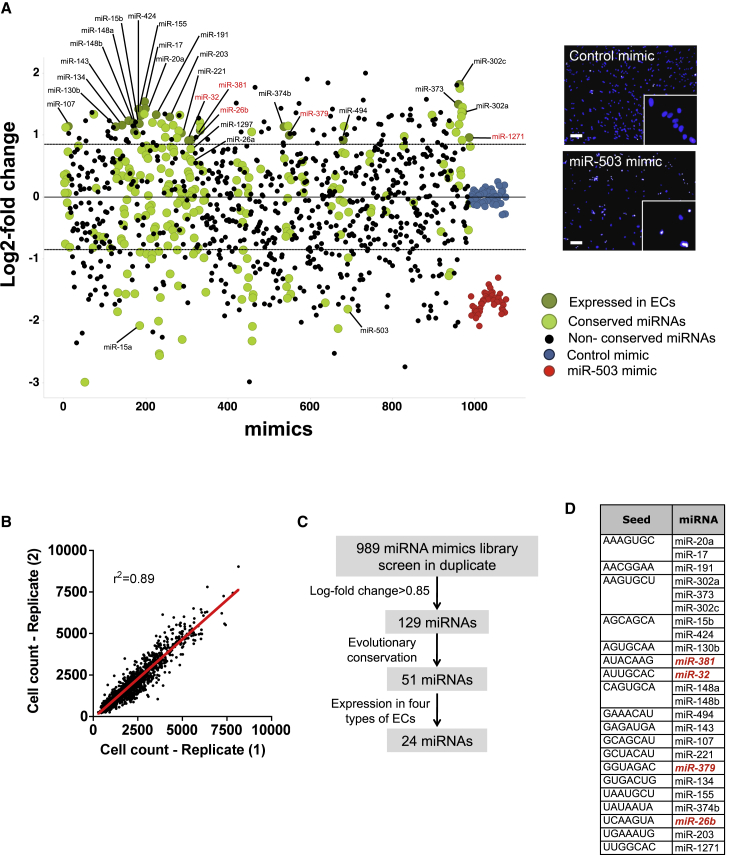
Phenotypic Screen of miRNAs Regulating HUVEC Growth (A) Left panel: log2 (fold change of miR mimics versus control mimics) values of cell count on the y axis is plotted against miR-mimics on the x axis. Non-conserved miRNAs (black), conserved miRNAs (light green), and miRNA enhancers of cell growth that are conserved and expressed in endothelial cells (dark green) are shown. Positive controls, miR-503 mimic, red; negative controls, miR-control mimic, blue; black dashed lines, cutoff log2 values in either direction >0.85 or <−0.85. Right panel: representative images of negative and positive controls from a screen plate. Nuclei were stained with DAPI (blue). Scale bar, 100 μm. (B) Correlation of screen plate replicates for the raw data of cell count parameter (r^2^ = 0.89). (C) Schematic describing the filtering and selection process of hit enhancers. (D) The selected hit enhancers are listed with their seed region. These miRNAs are expressed among four different EC types: HUVECs, human coronary artery endothelial cells (HCAECs), human aorta endothelial cells (HAECs), and human dermal micro-vascular endothelial cells (HMVECs). miRNAs that have not previously been studied in the context of angiogenesis are in red.

We conducted a phenotypic high-throughput screening of a human miRNA mimic library for their effect on human umbilical vein EC (HUVEC) growth. The quality of the screen was assessed by measurement of the Z’ factor, which resulted in a technical Z’ factor of >0.3 per plate and overall Z’ factor of 0.3 (Figure S1). The screening results showed 129 miRNAs enhanced EC growth by more than 0.85 log fold change (equivalent to 1.8-fold at linear scale) when compared with the ECs treated with the miRNA controls, and 182 miRNAs reduced the EC growth (log2 fold change <−0.85) (Figure 1A). The screening was performed in duplicate; the replicates showed excellent reproducibility (r^2^ > 0.8; Figure 1B). Importantly, many of the well-characterized miRNAs regulating cell proliferation and/or angiogenesis, including miR-17, miR-221, and miR-302c, are included in our screening results.

We next concentrated on miRNAs enhancing EC growth. Based on data retrieved from small RNA sequencing datasets (Encyclopedia of DNA Elements [ENCODE]), we found 24 were both evolutionarily conserved and accounted for their expression in four different EC sources: HUVECs, human coronary artery endothelial cells (HCAECs), human aorta endothelial cells (HAECs), and human dermal micro-vascular endothelial cells (HMVECs) (Figures 1C and 1D). Among 24 miRNAs, five miRNAs (miR-32, miR-381, miR-26b, miR-379, and miR-1271) had not previously been reported to regulate EC growth activities and angiogenesis in the literature. miR-26b has the highest expression in ECs compared to the other four hits. Therefore, we chose miR-26b to focus on for validation and mechanistic follow-up studies.

### miR-26b Regulates EC Growth, Survival, and Tube Formation

**Figure 2:**
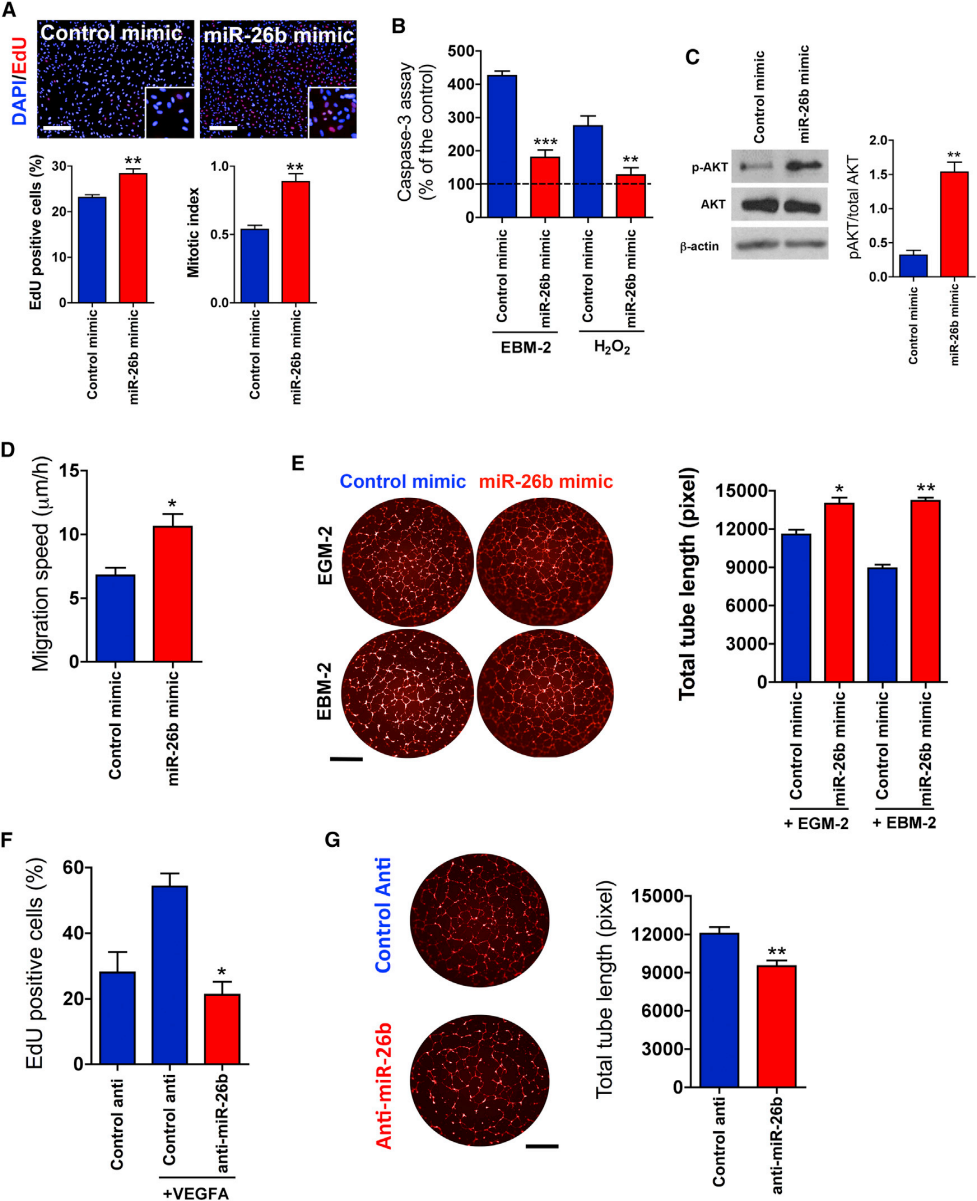
miR-26b Regulates EC Growth, Survival, Migration, and Tube Formation (A) Upper panel: representative images showing the effect of miR-26b mimics on cell number and DNA synthesis in HUVECs. Cells were fixed 72 hr after mimic transfection. Cells were stained for EdU (green) and DAPI (blue). Scale bars, 200 μm. Lower panel: quantitative data for the proportion of EdU-positive nuclei and mitotic cells to total cell number are shown. Mitotic cells are identified by their high content of EdU and DAPI staining. Mitotic index is the ratio between the number of cells in mitosis and the total number of cells. Error bars are mean ± SEM; **p < 0.01 compared to miR-control mimic (n = 6 replicates, unpaired t test). (B) Effect of miR-26b mimics on apoptosis induced by starvation (EBM-2) or H_2_O_2_ (500 μM). H_2_O_2_ was added after 48 hr of transfection for an additional 24 hr. Measured caspase-3 activity is normalized to controls and is expressed as mean ± SEM; n = 4 per condition; one-way ANOVA followed by Bonferroni post hoc test, *p < 0.01 and **p < 0.001 compared to miR-control mimics. (C) Immunoblotting for phospho-AKT (p-AKT^S473^) and total AKT1 (65 kDa) detection in HUVECs after 72 hr of miR-26b mimic or miR-control mimic transfection. β-actin was detected as a loading control. (D) Effect of miR-26b mimic on HUVEC migration speed measured for 8 hr by electric cell-substrate impedance sensing. The assay was performed 72 hr after mimic transfection. Error bars are mean ± SEM; *p = 0.02 compared to miR-control mimic (n = 4 replicates, unpaired t test). (E) Left: representative images showing effect of miR-26b mimics on EC tube formation with vascular growth factor media (EGM-2) or without growth factor media (EBM-2). Scale bar, 1 mm. The assay was performed 72 hr after mimic transfection. Tubes were fixed after 8 hr and stained with phalloidin alexa 568. Right: total tube length (pixels) is expressed as mean ± SEM; n = 5 per condition; one-way ANOVA followed by Bonferroni post hoc test, *p < 0.01 and **p < 0.001 compared to miR-control mimics. (F) Effect of anti-miR-26b on DNA synthesis in HUVECs activated by VEGFA (10 ng/mL) during the experiment. Cells were fixed 72 hr after mimic transfection. EdU-positive cell percentage is expressed as mean ± SEM; n = 4 per condition; one-way ANOVA followed by Bonferroni post hoc test, *p < 0.01 compared to control anti-miR. (G) Effect of anti-miR-26b on EC tube formation. Representative bright-field images of tube formation for HUVECs transfected with anti-miR-26b and control anti. Scale bar, 1 μm. The assay was performed 72 hr after anti-miR transfection. Tubes were fixed after 8 hr and stained with phalloidin alexa 568. Error bars are mean ± SEM; **p < 0.01 compared to control anti-miR (n = 6 replicates, unpaired t test).

To confirm the effect of miR-26b on EC proliferation, we measured DNA synthesis using an EdU cell proliferation assay. We found that miR-26b significantly enhanced EdU incorporation and mitotic index in HUVECs (Figure 2A). Furthermore, miR-26b exerted a pro-survival effect on ECs under starvation for 24 hr or following exposure to H_2_O_2_ (Figure 2B). The pro-survival effect of miR-26b on HUVECs was confirmed by the increase of AKT1 phosphorylation level (Figure 2C). The proliferative and pro-survival effects of miR-26b were abolished when it was incubated with dominant-negative inhibition of AKT1 signaling (Figure S2).

Using electric cell-substrate impedance sensing (ECIS), we found that miR-26b overexpression increased EC migration speed (Figure 2D). The overexpression of miR-26b enhanced the EC tube formation and branching morphogenesis in full vascular growth factor media or reduced vascular growth factor media (Figure 2E). Next, we investigated the effect of miR-26b inhibition using anti-miRs on EC growth and tube formation, and we found the anti-miR-26b decreased both VEGF-driven EC growth and tube formation (Figures 2F and 2G). We determined whether the endogenous expression of its family member miR-26a is affected by the miR-26b overexpression or inhibition, and we found that the expression level of miR-26a is not affected by the modulation of miR-26b expression (Figure S3). Our observations reveal that, *in vitro*, miR-26b regulates EC growth, survival, migration, and tube formation.

### Identification of miR-26b Gene Target-Mediated EC Growth and Survival

**Figure 3:**
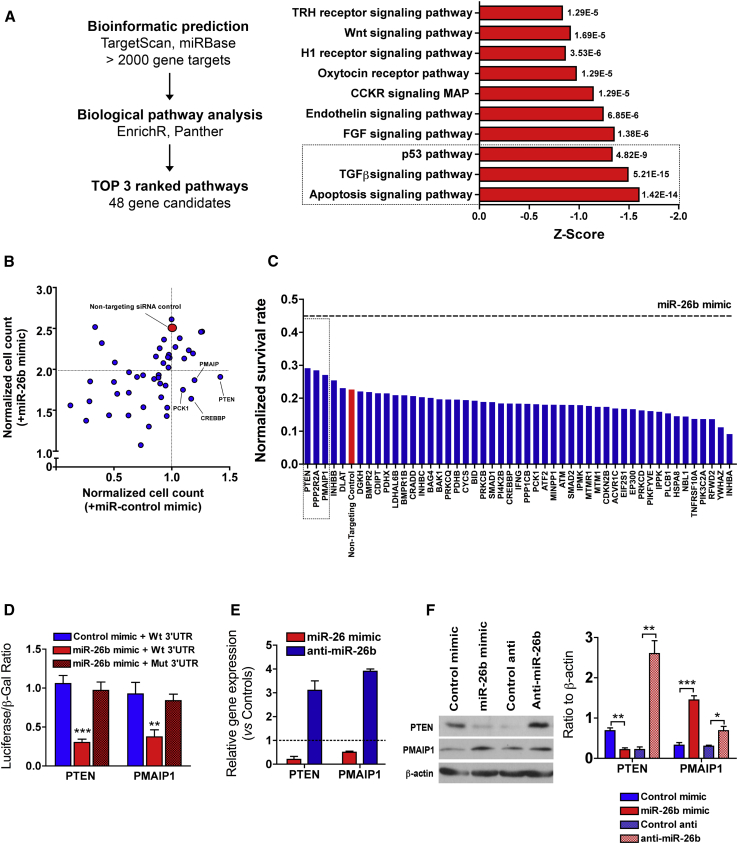
Identification of miR-26b Target Genes (A) Workflow for miRNA target identification (left panel). Genes were classified into molecular function according to the PANTHER classification system using EnrichR open source. Signaling pathways identified by the PANTHER classification system were plotted based on *Z* scores within each pathway (right panel). (B) siRNA-transfected HUVECs co-transfected with miR-control or miR-26b mimics. Cell count was captured 72 hr later. Cell count was normalized to non-targeting siRNA control-/miR-control mimic-transfected cells. Horizontal dashed line shows the cutoff value <2-fold for target genes abolishing the effect of pro-proliferative of miR-26b mimic. (C) siRNA-transfected HUVECs were incubated without or with a high dose of H_2_O_2_ (500 μM) for 24 hr. Horizontal dashed line shows the survival rate level of miR-26b mimic-transfected cells. Non-targeting siRNA control, red. (D) Luciferase gene reporter assays in HEK293 cells confirmed miR-26b binding to wild-type (WT) 3′ UTR or mutated (mut) 3′ UTR of PTEN and PMAIP1. Data are shown as the mean ± SEM of 6 independent experiments; **p < 0.01 and ***p < 0.001 versus miR-control mimic. Two-way ANOVA followed by Bonferroni post hoc test. (E) HUVECs were transfected with miR-26b mimic, anti-miR-26b, miR-control mimic, or anti-miR-control. At 3 days post-transfection, RNA was extracted and the levels of PTEN and PMAIP1 were determined by qRT-PCR. Values were normalized to S18 and then to the controls (mimic or anti-miR). Data are shown as the mean ± SEM of 4 independent experiments (*p < 0.01). (F) Cells were lysed and the expression of PTEN and PMAIP1 were analyzed by immunoblotting. β-actin was detected as a loading control.

It is important to note that identifying functionally important miRNA targets is crucial for understanding miRNA functions. Bioinformatic analysis of miR-26b targets revealed more than 2,000 predicted gene targets. Consequent functional annotation analysis of predicted target genes revealed enrichment for genes belonging to FGF-, transforming growth factor β (TGF-β)-, p53-, and apoptosis-signaling pathways, which are related to cell growth and survival functions (Figure 3A). Among the genes in these, we focused on 48 gene candidates selected from the top-ranked pathways to study their potential regulation by miR-26b. We applied two phenotypic loss-of-function approaches to prioritize the gene targets using a siRNA screen of 48 gene target candidates. First, to determine which gene target mediates the pro-proliferative effect of miR-26b, siRNA-transfected HUVECs were co-transfected with miR-26b or miR-control mimic. We found that four siRNAs (PTEN, PMAIP1, PCK1, and CREBBP) that prevented miR-26b induced more than two folds of EC growth (Figure 3B). Second, we investigated the siRNA-transfected HUVECs on the survival rate in the presence of H_2_O_2_ exposure for 24 hr. We identified three genes (PTEN, PPP2R2A, and PMAIP1) that enhance EC survival rate with similar potency compared with miR-26b (Figure 3C).

PMAIP1 (also known as Noxa) and PTEN resulted from the two functional assays, and both negatively regulate cell survival.^31^, ^32^ PPP2R2A was excluded from further analysis because it was not regulated at an mRNA or protein level by miR-26b mimics or anti-miR-26b (Figure S4). To further validate direct miRNA binding in the 3′ UTR of PTEN and PMAIP1, we used the luciferase reporter vector system as described before,^21^ in which 3′ UTR luciferase plasmids of PTEN and PMAIP1 were co-transfected with miR-26b mimic or its control mimics into HEK293 reporter cells. We were not able to perform luciferase gene reporter assays in ECs because of low transfection efficacy. Luciferase activity was significantly lowered when miR-26b was transfected compared with control mimic treatment, whereas mutation of the binding sites restored it (Figure 3D). We validated that miR-26b mimics downregulated the mRNA expression of the two target gene candidates, whereas anti-miR-26b increased their expression (Figure 3E). Western blot analysis demonstrated that only PTEN is regulated at a protein level at 72 hr after transfection with miR-26b mimic or anti-miR-26b (Figure 3F), whereas PMAIP1 is downregulated by miR-26b mimic only at 16 hr after transfection and accumulated at the same control levels after 48 hr (Figure S5). Thus, we found that PTEN in ECs is the most suitable target among the selected genes.

### miR-26b Promotes *In Vivo* Microvascular Growth

**Figure 4:**
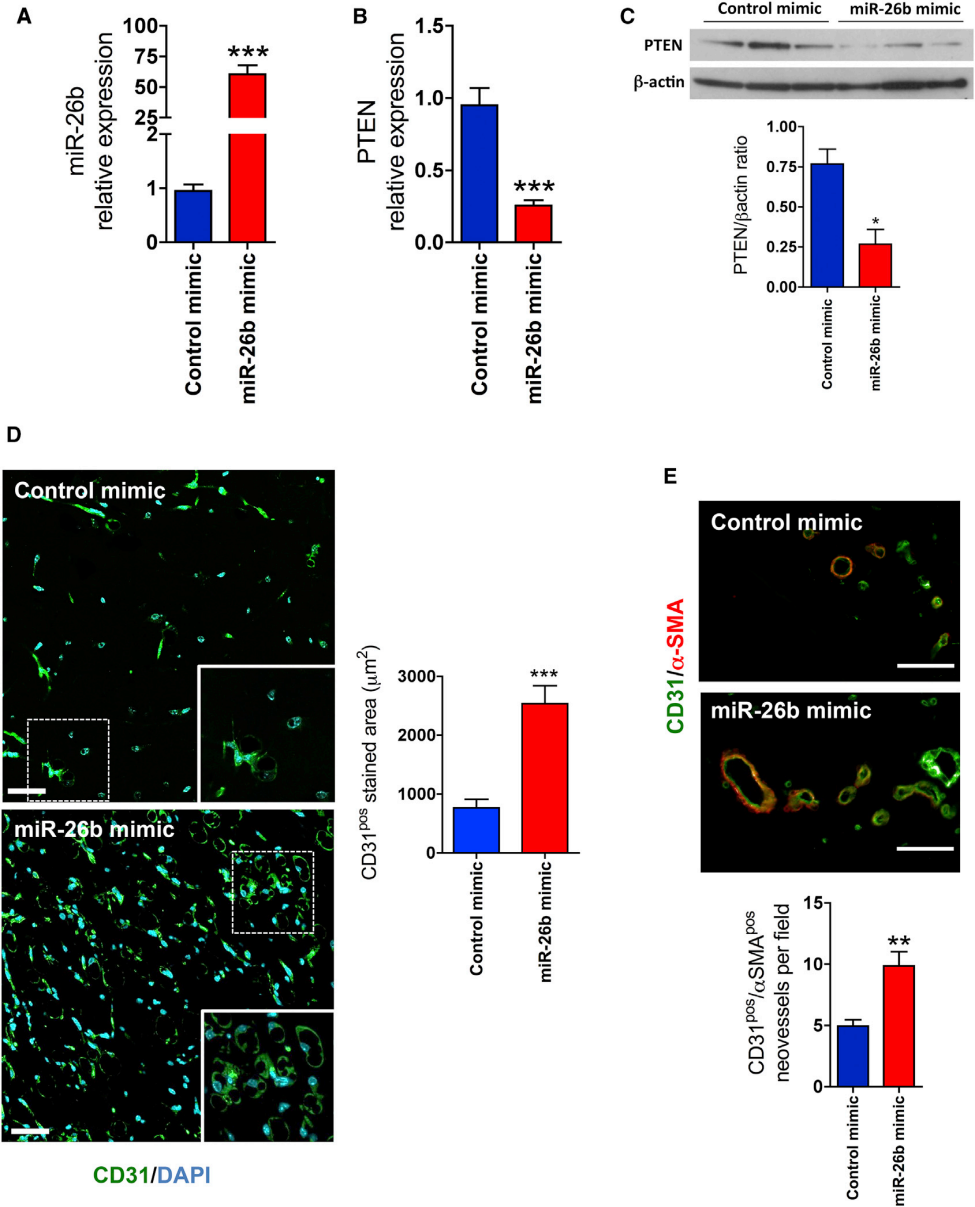
miR-26b Promotes Microvascular Growth *In Vivo* (A and B) The expression of (A) miR-26b and (B) PTEN in Matrigel plug mixed with miR-26b mimic and control mimic after 12 days. (C) Western blot analysis of PTEN expression in Matrigel plugs. β-actin was detected as a loading control. Lower panel: quantification of western blotting is shown. Error bars are mean ± SEM; *p < 0.05 compared to controls (n = 3 animals per group, unpaired t test). (D) Left panels: representative images showing the new microvessels positive for CD31 (green), with the predominant linear structures indicating small vessels and some apparently circular structures indicating larger vessels in the implanted plugs. Scale bars, 50 μm. Right panel: quantification of the area of CD31 coverage in the Matrigel plugs mixed with miR-26 mimic or control mimic at 12 days after implantation is shown. (E) Upper panel: representative images of Matrigel plugs showing structures double positive for green (CD31) and red (α-SM actin) indicate vessels with mural cell coverage, which are more mature vessels. Scale bar, 25 μm. Lower panel: quantification of the number of double-positive CD31 and α-SM actin vessels per field in the Matrigel plugs mixed with miR-26 mimic or control mimic at 12 days after implantation is shown. Error bars are mean ± SEM; **p < 0.01 and ***p < 0.001 compared to controls (n = 6 animals per group, unpaired t test).

To investigate whether miR-26b has pro-angiogenic properties *in vivo*, miR-26b mimic and miR-control mimic were incorporated in the Matrigel and injected subcutaneously into the mice. After 12 days, mimics regulated miR-26b expression in the plugs (Figure 4A), resulting in the opposite expression of its identified target gene PTEN at protein and mRNA levels (Figures 4B and 4C). Matrigel plugs mixed with miR-26b mimic exhibited more neovascularization, characterized by the increase of CD31-stained area, than those mixed with control mimic (Figure 4D). The quantification of mature microvessels, determined on the basis of pericyte or smooth muscle cell-covered endothelium (vascular structures that are double positive for α-smooth muscle [SM] actin and CD31), confirmed that miR-26b mimic enhances the induction of mature vasculature (Figure 4E). This suggests that, *in vivo*, forced overexpression of miR-26b enhances the microvascular growth and mature neovascularization.

### miR-26b Increases *In Vivo* EC Survival following Acute Ischemia

**Figure 5:**
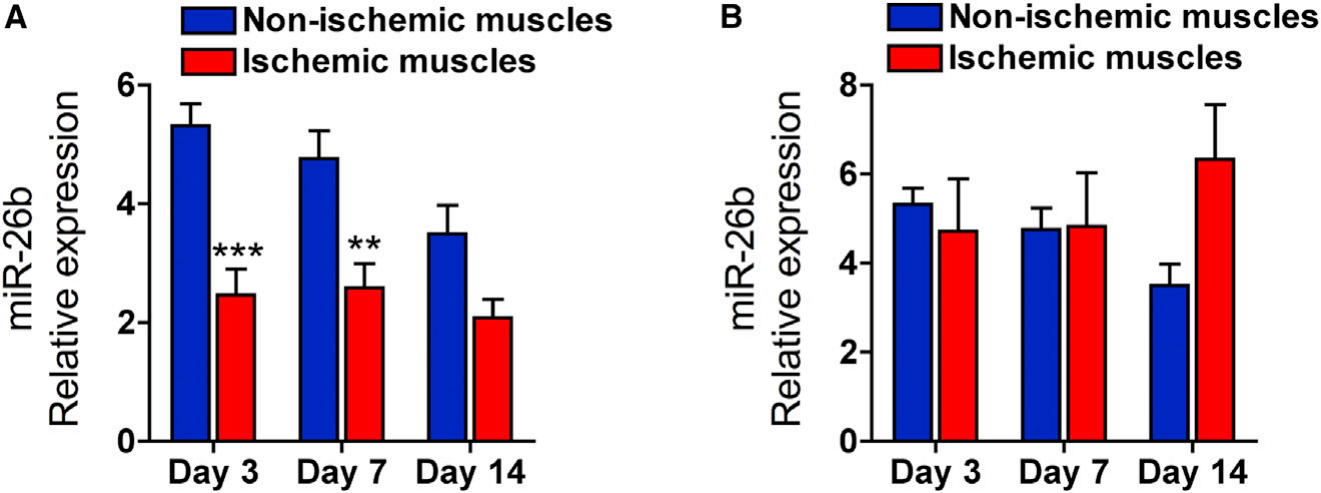
Time Course of Endogenous miR-26b Expression in the Adductor Muscle following Ischemia Injury (A and B) Expression of miR-26b in (A) endothelial cell (CD31^pos^/CD45^neg^) fraction and (B) muscle fraction isolated from the adductor muscles, which were collected at 3, 7, and 14 days after ischemia induction. miR-26b levels were normalized against snRU6 control. Error bars are mean ± SEM; **p < 0.01 and ***p < 0.001 compared to controls (n = 6 replicates, unpaired t test).

To assess the endogenous expression of miR-26b level in response to ischemic insult, ECs were isolated from mouse adductor muscles at 3, 7, and 14 days following the induction of limb ischemia. The EC population was sorted based on the high expression of CD31 and the lack of CD45 expression. EC purity was confirmed by the co-expression of other constitutive EC markers, such as CD144 (VE-cadherin) and CD105 (endoglin) (Figure S6). The expression level of miR-26b in isolated ECs significantly decreased at 3 and 7 days post-ischemia compared with sham-operated controls (Figure 5A). Of note, the expression level of miR-26b after ischemia was altered only in the endothelium and it was not in the whole of ischemic muscle fraction (Figure 5B).

**Figure 6:**
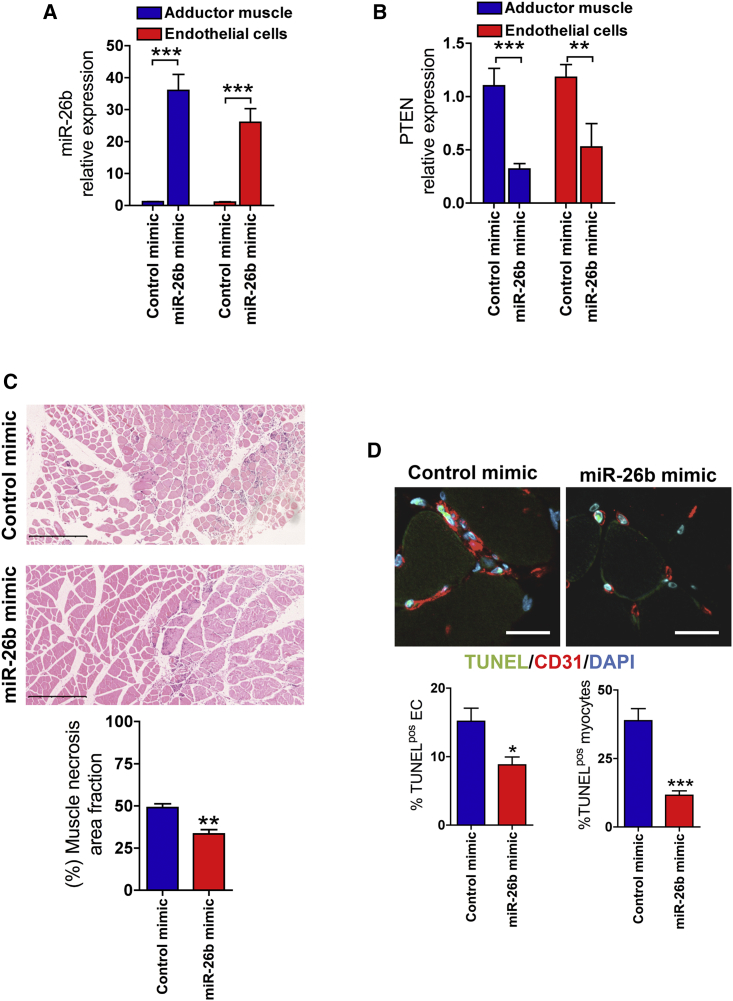
miR-26b Protects ECs and Myocytes from Ischemic Injury (A and B) Expression of (A) miR-26b and (B) PTEN in ECs sorted from adductor muscles and total adductor muscles 3 days after ischemia and intramuscular oligonucleotide delivery. Error bars are mean ± SEM; **p < 0.01 compared to controls (n = 5 animals per group, unpaired t test). (C) Representative images of H&E staining of ischemic adductor muscles injected with miR-26b mimic or control mimic. Scale bar, 500 μm. Lower panel: quantitative analysis of muscle necrosis extent in the ischemic adductor muscles injected with miR-26b mimic and control mimic (necrosis area was normalized to the total muscle area) is shown (n = 5 per group). Error bars are mean ± SEM; **p < 0.01 compared to control mimic (n = 5 animals per group, unpaired t test). (D) Representative images of TUNEL staining of ischemic adductor muscles injected with miR-26b mimics or control mimic. CD31, red; TUNEL staining, green; DAPI, blue. Scale bars, 30 μm. Lower panel: quantification of ECs or myocytes positive for TUNEL staining is shown. Error bars are mean ± SEM; *p < 0.05 and ***p < 0.001 compared to control (n = 5 animals per group, unpaired t test).

Next, we evaluated whether administration of miR-26b mimics would improve EC survival following ischemic injury. Recently the efficacy of different lipid formulations in delivering miRNA mimics has been confirmed in a mouse model of ischemia.^33^ Mice were subjected to hindlimb ischemia and followed by local administration of miR-26b or miR-control mimics, complexed with a lipid transfection reagent. At day 3 after injection, we first measured the expression levels of the injected miR-26b mimic and its target PTEN in the isolated ECs from adductor muscle and in total adductor muscle. We detected a significant increase in the levels of miR-26b in both ECs and total muscle (Figure 6A). This was associated with a significant decrease in the expression level of PTEN (Figure 6B), indicative of effective transfection and sustained activity of forced expressed miR-26b.

**Figure 7:**
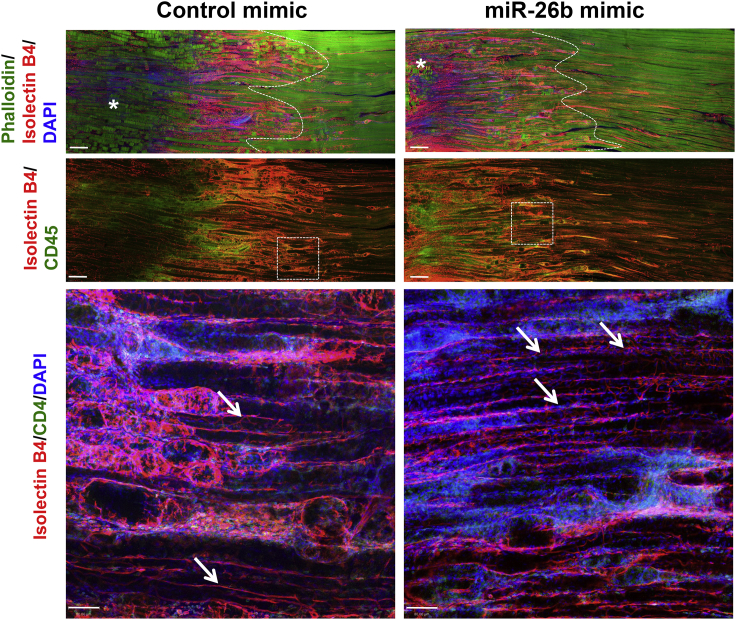
miR-26b Mimic Preserves the Native Microvasculature Network Morphology following Ischemia Injury Representative confocal microscopy images of thick longitudinal sections for the ischemic adductor muscles injected with control mimic (left panels) or miR-26b mimic (right panels) at day 3 after ischemia. Upper panel: effect of exogenous miR-26b mimic injection on the survival of native muscle fibers and microvasculature network is shown. Asterisks denote the necrotic (dead) muscle fibers. Dotted line delimits the border of necrotic area. Myocytes (phalloidin, green), microvessels (isolectinB4, red), and nuclei (DAPI, blue) are shown. Scale bar, 340 μm. Middle panel: effect of exogenous miR-26b mimic injection on the native microvasculature network and the infiltration of leukocytes in the ischemic muscles is shown. Leukocytes (CD45, green) and microvessels (isolectinB4, red) are shown. Scale bar, 340 μm. Lower panel: inset of a higher magnification shows the microvascular network architecture in the necrotic area of ischemic adductor muscle. Arrowheads point to the preserved native microvasculature network in the necrotic areas. Leukocytes (CD45, green), microvessels (isolectinB4, red), and nuclei (DAPI, blue) are shown. Scale bar, 80 μm.

Histological examination of muscle cross-sections revealed less necrotic areas in the ischemic muscles treated with miR-26b mimic compared to control (Figure 6C). The pro-survival effect of miR-26b on ECs and myocytes was confirmed by terminal deoxynucleotidyl transferase dUTP nick end labeling (TUNEL) staining. We found that the overexpression of miR-26b resulted in a significant reduction of apoptosis in both ECs and myocytes (Figure 6D). We assessed the effect of miR-26b overexpression on the survival of native microvasculature network structure following ischemia injury. Confocal three-dimensional (3D) imaging exhibited a better preservation of native microvasculature network integrity in the necrotic area of ischemic adductor muscles treated with miR-26b mimic compared to the controls (Figure 7).

Next, we tested whether the local overexpression of miR-26b mimic could affect the remodeling of collateral arterioles in the ischemic adductor muscles, and we found no difference in the total area of α-SM actin-positive collateral vessels between miR-26b mimic-treated adductor muscle and the controls after 3 days of ischemia injury (Figure S7A). Moreover, both the miR-26b mimic-treated group and control mimic-treated group showed no difference in foot perfusion recovery during the 3 days following femoral excision (Figure S7B). Collectively, these results show that local overexpression of miR-26b enhances the survival of native microvasculature network following ischemia injury, which is associated with less muscle fiber necrosis.

## Discussion

Cell proliferation is stimulated only in vascular ECs needed for the growth of new blood vessels or vascular repair after injury. Using a high-throughput screening approach of an miRNA mimic library, we found many miRNAs positively and negatively modulate the EC growth. For instance, miR-17 and miR-221 were identified as enhancers of EC growth. A previous study showed that VEGF-mediated upregulation of miR-17 expression was necessary for *in vitro* EC proliferation and angiogenic sprouting. Furthermore, *in vivo* specific deletion of miR-17 in endothelium has reduced physiological retinal angiogenesis during development and diminished VEGF-induced tumor angiogenesis.^34^ miR-221 has been shown to be required for endothelial tip cell proliferation and migration. Exogenous miR-221 expression increased bromodeoxyuridine (BrdU) incorporation in cells that contributed to the dorsal longitudinal anastomotic vessels in the zebrafish.^19^ Our phenotypic screening revealed that miRNAs, such as miR-15a, miR-24, and miR-503, decreased EC growth significantly. These miRNAs have been previously studied in EC biology, and they demonstrated their deleterious effect on EC proliferation and angiogenesis.^21^, ^35^, ^36^

### miR-26b Is an Enhancer of Endothelial Cell Growth

Among miRNA hits, miR-26b was identified as a novel candidate that enhances EC proliferation and survival in several relevant *in vitro* and *in vivo* models. miR-26b is a member of the miR-26 family, which is localized on chromosome 2. The other members of the family constitute miR-26a-1, localized on chromosome 3, and miR-26a-2, localized on chromosome 12. Their mature products share an identic sequence.^37^ It only differs from the mature miR-26b sequence by 2 nt. They are predicted to target nearly identical sets of genes. However, it seems that the miR-26a isoform has a different role in EC proliferation and angiogenesis. Indeed, it has been shown that overexpression of miR-26a induced cell-cycle arrest and inhibited the *in vitro* angiogenesis through targeting smad1 3′ UTR. The systemic administration of LNA-anti-miR-26a increased angiogenesis, improved heart function following infarction, and decreased infarct size.^38^ A possible explanation for these discrepancies is that miR-26b levels vary significantly in response to an angiogenic stimulus, suggesting that growth conditions influence miRNA function in cultured cells.

Upon the stimulation of ECs by TNF-α or VEGF, the expression levels of both miR-26a and miR-26b were downregulated distinctively.^38^ In other primary vascular cells, miR-26a promotes smooth muscle cell proliferation via the TGF-β-signaling pathway. Conversely, the inhibition of miR-26a enhanced a contractile phenotype of smooth muscles.^39^ Our findings and those of others show that the actions of miR-26b are not shared by miR-26a, which would be valuable to examine in future vascular studies.

Previous studies have demonstrated that the expression level of miR-26b is reduced in the tissues of many cancers of liver,^40^ breast,^41^ and colon.^42^ Functionally, miR-26b exerts a tumor-suppressive role in many types of cancer, and the miR-26b-mediated growth inhibition is achieved through suppression of target genes like OCT4,^43^ SMAD1, CTGF,^44^ and/or COX2.^45^ In contrast, overexpression of miR-26a in a murine glioma model enhances *de novo* tumor formation.^37^ These observations raise the possibility that the miR-26 family governs context-specific changes in endothelial behavior depending on cell type or tissue microenvironment. Thus, while miR-26b plays an important pro-proliferative and survival signal during angiogenesis, it may play different roles in cancer cell growth.

### Validation of miR-26b Targets

The phenotypic approach can be used for miRNA target identification, recognizing the targets that are relevant for a specific phenotype. This is particularly important because one miRNA can have hundreds of putative targets, but only a few of these might be accountable for the phenotype under investigation. In this study, by combining target prediction- and functional RNAi-screening approaches, we have prioritized two gene target candidates, PTEN and PMAIP1, that could be responsible for the proliferative and survival phenotype induced by miR-26b. Within the 3′ UTR region of both mRNA targets, there are potential binding sites for miR-26b, and accordingly, we detected a robust downregulation of mRNA levels of the two target genes.

PMAIP1 has been shown to be a critical mediator of apoptotic signaling, and it functions primarily by inactivating the anti-apoptotic Bcl-2 family protein Mcl-1.^46^ Elevated PMAIP1 levels were detected after transient ischemia experiments *in vivo*, and delivery of PMAIP1 antisense oligonucleotides significantly reduced infarct volumes of rat brains.^47^ It seems that PMAIP1 is highly regulated at the transcriptional and post-transcriptional levels.^48^ In our study, a discrepancy between mRNA and protein levels for PMAIP1 was detected. This is in line with the post-translational modification and protein stability observed previously for this protein.^49^, ^50^ The induction of a survival program by miR-26b could lead to an increase in the protein stability of cellular PMAIP1 without consequence on its pro-apoptotic activity. Therefore, it remains unclear whether the transcriptional downregulation of PMAIP1 by miR-26b is sufficient to contribute to its survival effect on ECs. PTEN possesses lipid phosphatase activity that functions as a direct antagonist of PI3K- and AKT1-dependent signaling. Notably, the PI3K-AKT1-mTOR pathway is involved in the regulation of constitutive PMAIP1 levels in cancer cells, and its inhibition has an impact on the accumulation of PMAIP1 protein.^49^ With this in mind, the downregulation of PTEN due to miR-26b mimic and the consequent activation of the AKT1/mTOR pathway could be responsible for the accumulation of PMAIP1 in our experiments.

The inhibition of endogenous endothelial PTEN in cultured ECs potently enhances a variety of VEGF-mediated cellular responses, including cell survival and migration.^51^ In agreement with our findings, PTEN has been previously characterized as a direct target of miR-26b in different cell types.^37^, ^52^, ^53^, ^54^ Our study highlights that the survival effect of miR-26b on ECs is mediated mainly by regulating the expression of PTEN.

### *In Vivo* Effects of miR-26b Overexpression

In our study, the delivery of miR-26b mimics resulted in a high level of miR-26b expression in both animal models: Matrigel plug assay and limb ischemia. Moreover, the overexpression of miR-26b was associated with the inhibition of its identified target expression of PTEN. In agreement of *in vitro* functional assays, miR-26b overexpression enhances the microvascular growth and angiogenesis in the Matrigel plug model. Interestingly, following ischemia injury, endogenous expression of the miR-26b level is decreased in the endothelium of skeletal muscles, while the forced local overexpression of miR-26b in the adductor muscles improves the survival of ECs and muscle fibers in the acute phase. In particular, miR-26b maintains an intact structure of the native capillary network, as revealed by the examination of the 3D structure of microvasculature. As a consequence of preserved native microvasculature network, it is expected that muscle fibers would be more tolerant to hypoxia and, thereby, exhibit less necrosis and inflammatory infiltrate.^55^, ^56^ Nevertheless, we cannot exclude a direct effect of miR-26b mimic on the survival of muscle fibers following ischemic injury, since the overexpressed miR-26b was detected in the muscle fraction. In our study, we found that a high level of miR-26b resulted in an inhibition of PTEN expression in the ischemic adductor muscles. At the molecular level, we can speculate that PI3K activation may occur via a loss of PTEN and, subsequently, blunt the adverse effect of ischemia on endothelium and muscle fibers.

Adductor muscles accommodate collateral vessels, which undergo a growth and remodeling process (arteriogenesis) to compensate for the lack of blood flow in the acute phase (2–7 days) after ischemia.^57^ In our study, miR-26b overexpression in the adductor muscle did not affect the arteriogenesis, and no change in foot perfusion recovery was detectable using a laser speckle contrast imaging strategy. Further investigations are warranted to fully characterize the effect of miR-26b overexpression on angiogenesis and blood flow recovery in the chronic phase of limb ischemia.

### Conclusions

High-content screening has been used as a functional discovery tool to identify the miRNAs related to cellular phenotypes.^29^, ^58^ Gain-of-function genetic screens are well-established methods to identify genes sufficient to confer a particular cellular phenotype.^59^ The use of a gain-of-function approach recapitulates the situation where the level of a particular miRNA is enhanced following the physiological or pathological stimulus. In our study, using this discovery tool, the role of miR-26b was highlighted in the EC growth, survival, and angiogenesis. Administration of miR-26b mimic could be a promising therapeutic approach for the ischemic vascular disease. Future phenotypic screenings of miRNA function using angiogenesis assays will likely reveal novel roles for miRNAs in vascular formation, function, and homeostasis.

## Materials and Methods

### High-Content Screening of the miRNA Library

Pooled primary HUVECs were purchased from Lonza. Cells were maintained in EC medium (EGM-2, Lonza). HUVECs were reverse transfected in duplicate with a library of miRNA mimics (989 mature miRNAs with sense sequences, miRIDIAN microRNA Mimic Library, Dharmacon) in cell carrier 384-well flat clear-bottom black plates (PerkinElmer), using a standard reverse transfection protocol. Briefly, HUVECs (1,000 cells/well in 21 μL complete EGM-2 media) were seeded into a well containing 14 μL transfection mix (6.93 μL Optimem, 0.07 μL RNAi Max (Invitrogen), and 7 μL 120 nM miRNA), giving a final mimic concentration of 24 nM. 24 hr later, cells were incubated with 35 μL fresh EGM-2 media for an additional 48 hr at 37°C. At the 72-hr endpoint, cells were washed, fixed, and stained with DAPI dye. All liquid-handling steps, including seeding, fixation, washing, and staining, were performed using a Janus robotics (PerkinElmer). miR-503 mimic (inhibitor of EC growth)^21^ and non-targeting-control miRNA mimic were included with each screening plate as positive and negative controls, respectively.

Plates were imaged automatically at 10× magnification using the high-content imaging system (Operetta). Nine identically positioned fields were acquired from each well (covering the whole well area). Quantification of the cell number was performed automatically using Harmony imaging analysis software. Positive and negative controls were served to measure Z’ factor that reflects the quality of screen.^60^ Cell number was normalized to non-targeting mimic miR-control of each plate to allow for the inter-plate comparisons. The data from replicates were then averaged and log2 transformed to show the fold change. The selection cutoff for enhancer or inhibitor hits $\left(h_{e/i}\right)$ was determined as follows,

$$h_{e/i}=\mu_{neg}\pm 10\sigma ,$$

where $\mu_{neg}$ is the mean of negative controls (non-targeting control mimic and σ is the SD of the negative controls. The calculated cutoff for hit enhancer or inihibitor was equivalent to ±1.8-fold at linear scale and ±0.85 at log2 scale.

### Cell Transfection and Transduction

Lipofectamine RNAiMAX (Thermo Fisher Scientific) was used to transfect HUVECs with miR-control mimic, miR-26b mimic, or anti-miR-26b (24 nM final concentration). Adenoviral particles of dominant-negative AKT1 (DN-AKT1) and EGFP (control vector) were produced and used as previously described.^61^

### EdU Cell Proliferation Assay

DNA synthesis was assessed using Click-iT EdU Alexa Fluor 488 HCS kit (Thermo Fisher Scientific), according to the manufacturer’s protocol. Briefly, EdU-labeling medium (10 μM final concentration) was added to miRNA-transfected HUVEC culture and incubated for 6 hr before fixation. Next, the cultured cells were fixed with 4% paraformaldehyde and treated with 0.1% Triton X-100 for 15 min at room temperature. After washing with PBS, the samples were stained with Click-iT reaction cocktail working solution at room temperature for 30 min. The cells were stained with DAPI at room temperature for 20 min. The plates were imaged and quantified using high-content fluorescent microscopy (Operetta). The total cell number and percentage of EdU-positive cells were calculated from 9 fields/well using a 10× objective (Harmony software, PerkinElmer).

### EC Migration Assay

Confluent HUVECs were transfected with controls or miR-26b mimic and plated on the ECIS chip array (8W1E) (Applied Biophysics). The migration speed was calculated in micrometers per hour as previously reported.^62^

### Caspase-3/7 Assay

Caspase-3/7 activity was measured using Caspase Glo 3/7 assay (Promega) according to the manufacturer’s protocol.

### Endothelial Tube Formation Assay

Matrigel (50 μL/well) was added to the wells in a 96-well plate and allowed to polymerize at 37 °C for 30 min. HUVECs (15,000 cells) previously transfected with miR-26b mimic, anti-miR-26b, or miR-control mimic were added to the top of the Matrigel. After incubation for 8 hr, tube formation was assessed by high-content imaging using the Operetta system. Total tube length and branching points were assessed by Metamorph image analysis software.^63^

### Bioinformatics for miR-26b Gene Target Prediction

Predicted miR-26b target-binding sites were obtained from TargetScan Human version (v.)7.0 (http://www.targetscan.org) and miRBase (http://www.mirbase.org). The prediction is based on scoring parameters such as context and conservation. Potential targets of miR-26b involved in cell growth- and survival-signaling pathways were identified using EnrichR online open source with Panther tool (http://amp.pharm.mssm.edu/Enrichr/).

### siRNA Screening to Validate Predicted Gene Targets of miR-26b

HUVECs were reverse transfected in duplicate with a cherry-pick library of 48 siRNAs (OFF-Target Smartpool, Dharmacon) of predicted target gene candidates for miR-26b in 96-well plates. For cell growth assay, HUVECs were co-transfected with miR-26b or miR-control mimics after 24 hr. Cells were then incubated for an additional 72 hr. Next, cells were fixed and stained with DAPI. The plates were imaged and quantified using high-content fluorescent microscopy (Operetta). The total cell number was calculated from 9 fields/well using a 10× objective (Harmony software, PerkinElmer). Averaged cell count for each well (siRNA) was normalized to non-targeting siRNA and miR-control mimic well condition. For EC survival assay, after 48 hr of siRNA transfection, transfected HUVECs were exposed to H_2_O_2_ (500 μM) for an additional 24 hr. Next, cells were fixed, stained, and imaged as described above. Averaged cell count for each well (siRNA) exposed to H_2_O_2_ was divided by the siRNA control condition (without H_2_O_2_ exposure). Then, values were multiplied by normalized cell count to non-targeting siRNA control. Both siRNA screens were performed in duplicate.

### 3′ UTR Luciferase Assay

A luciferase assay was performed as previously described.^21^ Empty 3′ UTR, PTEN 3′ UTR, and PMAIP1 3′ UTR vectors were from SwitchGear Genomic collection (Active Motif). We further validated the binding to PTEN and PMAIP1 using plasmids bearing the mutated version of the seed sequence in the 3′ UTR. For PTEN, we deleted the first 4,000 bp of the 3′ UTR sequence including the 4 binding sites. For PMAIP1, we mutated the single binding site in the 3′ UTR (Figure S8). Mutations were introduced using GeneArt Site-Directed Mutagenesis System (Thermo Fisher Scientific). Primers for 3′ UTR mutations were as follows: PTEN: forward 5′-ATGTGCAATAATGTAAAATATGAAG-3′, reverse 5′-GCACATTAGGACATGAGGGC-3′; and PMAIP1: forward 5′-TTACAAGAGTCTTATAACatatatatTTTTTAGTTAA-3′, reverse 5′-TTAACTAAAAAATATATATGTTATAAGACTCTTGTAA-3′. Luciferase constructs were co-transfected into HEK293T cells together with miR-26b mimic or miR-control mimic. p-SV-beta-Gal control vector was co-transfected in all conditions. Cells were cultured for 48 hr and assayed with the Luciferase and β-Galactosidase Reporter Assay Systems (Promega). Luciferase values were normalized to protein concentration and β-galactosidase activity.

### qRT-PCR and miRNA Detection

Total RNA was extracted using mini RNeasy kits or miReasy kit (QIAGEN). For mRNA analysis, cDNA was amplified by real-time qPCR and normalized to 18S rRNA. Each reaction was performed in triplicate. Quantification was performed by the 2^–**ΔΔ**Ct^ method.^64^ qPCR was used to measure PTEN, PMAIP1, PPP2R2A, and 18S rRNA. Primers were pre-designed from Sigma (KiCqStart Primers). Real-time quantification to measure miRNAs was performed with the TaqMan miRNA reverse transcription kit and miRNA assay (miR-26b) (Thermo Fisher Scientific) using Lightcycler 480 (Roche). miRNA expression was normalized to the U6 small nuclear RNA (snRU6).

### Western Blotting

Cells were lysed with radioimmunoprecipitation assay (RIPA) buffer (Sigma) mixed with protease cocktail inhibitor and phosphatase inhibitors (Roche), and protein concentration was determined with a bicinchoninic acid (BCA) assay (Pierce). Equal amounts of protein per sample were separated by SDS-PAGE and transferred to an Immobilon-P polyvinylidene fluoride (PVDF) membrane (Millipore). Membranes were blocked with 5% skim milk, followed by incubation with primary antibodies β-actin (Sigma; 1:1,000), PMAIP1 (Cell Signaling Technology; 1:1,000), PTEN, (Cell Signaling Technology; 1:1,000), PPP2R2A (Cell Signaling Technology; 1:1,000), total AKT1 (Cell Signaling Technology; 1:1,000), and S473 p-AKT (Cell Signaling Technology; 1:1,000) at 4 °C overnight. Appropriate horseradish peroxidase (HRP)-conjugated secondary antibodies were incubated for 1 hr at room temperature (RT) before the detection of protein with enhanced chemoluminescence reagents (Millipore). Western blots were quantified using ImageJ software (NIH).

### *In Vivo* Matrigel Plug Assay

Experiments involving mice were covered by project and personal licenses issued by the UK Home Office, and they were performed in accordance with the Guide for the Care and Use of Laboratory Animals (the Institute of Laboratory Animal Resources, 1996) and in accordance with Animal Research Report of *In vivo* Experiments (ARRIVE) guidelines. CD-1 mice (male, 10 weeks old) were subcutaneously injected into the groin regions of mice with 400 μL Matrigel containing recombinant mouse basic FGF (bFGF) (PeproTech, 250 ng/mL) and heparin (Sigma, 50 U/mL) mixed with miR-mimic control or miR-26b mimics (lipids [Lipofectamine RNAiMAX reagent, ratio 1:1 in volume] 5 μg/gel, n = 6 per group). After 12 days, mice were sacrificed, and the Matrigel plugs were removed and fixed in 4% paraformaldehyde. Paraffin cross-sections of plugs were deparaffinized and rehydrated. Antigen retrieval was performed by exposure to Proteinase K (Roche). The slides were blocked by incubating with 5% normal goat serum for 30 min, incubated with anti-CD31 primary antibody (Abcam; 1:200) overnight at 4°C, and then incubated with Alexa 488-conjugated anti-rat immunoglobulin G (IgG) antibody (Thermo Fisher Scientific) and α-SM actin-cy3 (Sigma; 1:250) for 1 hr at room temperature. Sections were analyzed and photographed using a fluorescence microscope. CD31-positive neovessel area covered with α-SM actin inside plugs was quantified using ImageJ software (NIH). Microvessel density was quantified in 25 fields/plug at each of three 1-mm-spaced sectioning planes ofplugs. CD31-positive neovessel area was quantified using ImageJ software (NIH). Microvessel density is expressed per square micrometer.

### Hindlimb Ischemia Model

Hindlimb ischemia was induced in mice as previously described.^65^ Briefly, 12-week-old male CD-1 mice (Charles River Laboratories) were anesthetized with Isofluoran. The left femoral artery was ligated through a skin incision. The artery was then ligated to the external iliac artery at the distal point where it bifurcates into the saphenous and popliteal arteries. The femoral artery between two point ligations was then electro-coagulated. Mice were sacrificed and the adductor muscles were harvested for further analysis.

### Isolation of Endothelial Cells from Mouse Limb Muscles

Ischemic and non-ischemic adductor muscles (n = 6 per group) were harvested at 3, 7, and 14 days after ischemia. Next, muscles were rinsed and digested with collagenase II (Worthington) plus DNase I (Sigma) using gentleMACS Dissociator, following the manufacturer’s protocol. Next, ECs were immunomagnetic sorted using a CD31 and CD45 (Miltenyi Biotech) as previously reported.^66^ RNA was extracted from EC fraction and muscle fraction for performing subsequent qPCR to measure the expression level of miR-26b, as described above. Purity of EC preparations was confirmed by flow cytometry using a cocktail of specific antibody markers for ECs: CD31 (Miltenyi Biotech; 1:50), CD144 (eBioscience; 1:50), and CD105 (eBioscience; 1:50).

### Local Delivery of miRNA Mimics into Adductor Muscles

Of a mixture containing miR-26b mimic (1 μg) or miR-control mimic (1 μg), lipofectamine RNAiMAX regaent (Thermo Fisher Scientific, 10 μL), and Opti-MEM (Thermo Fisher Scientific, 10 μL), 20 μL was injected into ischemic adductor muscles using a 0.3-mL insulin syringe with a 30G needle, as previously published.^33^ At 3 days after surgery, mice were sacrificed, and the injected adductor muscles were harvested for further histology (n = 5 per group) or RNA analysis (n = 4 per group).

### Laser Speckle Contrast Imaging of Blood Flow

Blood flow in the mouse paw was monitored using a Speckle Contrast Imager FLPI-2 (Moor Instruments, UK). The FLPI measurements were made in a warm (24°C) and quiet environment. The charge-coupled device (CCD) camera was positioned 30 cm above the mouse paw. The contrast images were processed to produce a color-coded live flux image (red denoted high perfusion, blue signified low perfusion) using the moorFLPI-2 measurement module (Moor Instruments). Measurements were made in the paws area of non-ischemic and ischemic sides before and immediately after limb ischemia induction. Additional measurements were acquired 24, 48, and 72 hr after ischemia.

### Histology, Immunostaining, and Morphometry

Ischemic adductor muscles were then removed and fixed with 4% buffered paraformaldehyde. Paraffin cross-sections were immunostained for ECs using anti-CD31 antibodies as described above. TUNEL staining (Promega) was performed to detect apoptosis, according to the manufacturer’s instructions. Next, the slides were co-stained with anti-CD31 for visualizing ECs, and the nuclei were further counter-stained with DAPI (Thermo Fisher Scientific). We used the auto-fluorescent signal of myocytes with their distinct morphology to identify them in the tissue. The TUNEL-positive nuclei of ECs and myocytes were semi-quantified using NIH ImageJ software. The area of necrotic tissues in the adductor muscle was analyzed by H&E staining. Necrotic cells display a more glassy homogeneous appearance in the cytoplasm with increased eosinophilia, whereas the nuclear changes are reflected by karyolysis, pyknosis, and karyorrhexis. Necrotic area was defined as the percentage of area, which includes these necrotic myocytes, inflammatory cells, and interstitial cells, compared to the total muscle area. In analyzing arteriogenesis, sections were stained with anti-CD31 and anti-α-SM actin as described above. Tiled images of the entire adductor muscle were acquired at ×10 for this analysis. The area of microvessels stained with α-SM actin, which were equal to or larger than 50 μm, were measured and normalized to the total adductor muscle area using ImageJ software (NIH).

### Whole-Mount Immunohistochemistry

At 3 days post-ischemia, anesthetized mice were perfused and fixed under physiologic pressure with 4% paraformaldehyde. Adductor muscles were isolated, carefully dissected under stereomicroscope, and snap frozen. Samples were cut into slices of 150-μm thickness. Briefly, following incubation with blocking buffer, the samples were incubated with primary antibody rat anti-mouse CD45 (Pharmagen; 1:100) overnight at 4°C. Further incubation with the appropriate secondary antibody, alexa 488-conjugated Isolectin B4 (Invitrogen; 1:50), alexa 647-conjugated Phalloidin (Invitrogen; 1:100), and DAPI (Invitrogen), was performed. Whole-mount muscle imaging was done on an LSM 710 Zeiss confocal microscope. Maximum-projection confocal images of the adductor muscle microvasculature were generated from z stacks (30–70 μm, 1- to 10-μm step size depending on specimen size, staining, and objective used) acquired starting at the medial surface of the adductor muscle specimens. To visualize large areas of the microvasculature network on the confocal microscope, a tile-scanning technique was employed whereby multiple overlapping (10% overlap) maximum-projection images were acquired with a 10× or 20× objective and a composite image was constructed by arraying the individual images using ZEN software (Zeiss).

### Statistical Analysis

Screen data were visualized using TIBCO Spotfire analytic software (PerkinElmer). Comparisons between different conditions were assessed using 2-tailed Student’s t test. Differences among groups were elicited using ANOVA statistical test followed by Bonferroni post hoc analyses as appropriate. Continuous data are expressed as mean ± SEM; a p value < 0.05 was considered statistically significant. Statistical analyses and graphics were performed using GraphPad Prism v.5.0.

## Author Contributions

A.M., D.M., A.C., and A.A. designed and conducted the *in vitro* experiments. M.M. and A.A. designed and conducted the *in vivo* experiments. A.H., D.E., and A.A. conducted the high-throughput screening. A.C. and A.A. directed the research and participated in the design and interpretation of experiments and the writing of the manuscript.

## Conflicts of Interest

The authors declare no conflicts of interest.

## Supplemental Information

Document S1. Figures S1–S8

Document S2. Article plus Supplemental Information
