# Supplementary material for: Phenotypic miRNA Screen Identifies miR-26b to Promote the Growth and Survival of Endothelial Cells
Source: Mol Ther Nucleic Acids. 2018 Aug 18;13:29–43. doi: 10.1016/j.omtn.2018.08.006 (PMC6141730; doi:10.1016/j.omtn.2018.08.006)
Supplement: Document S1. Figures S1–S8 [file mmc1.pdf]

OMTN, Volume *13*

Supplemental Information

Phenotypic miRNA Screen Identifies miR-26b to Promote the Growth and Survival of Endothelial Cells

Andrea Martello, David Mellis, Marco Meloni, Alison Howarth, Daniel Ebner, Andrea Caporali, and Ayman Al Haj Zen

A)

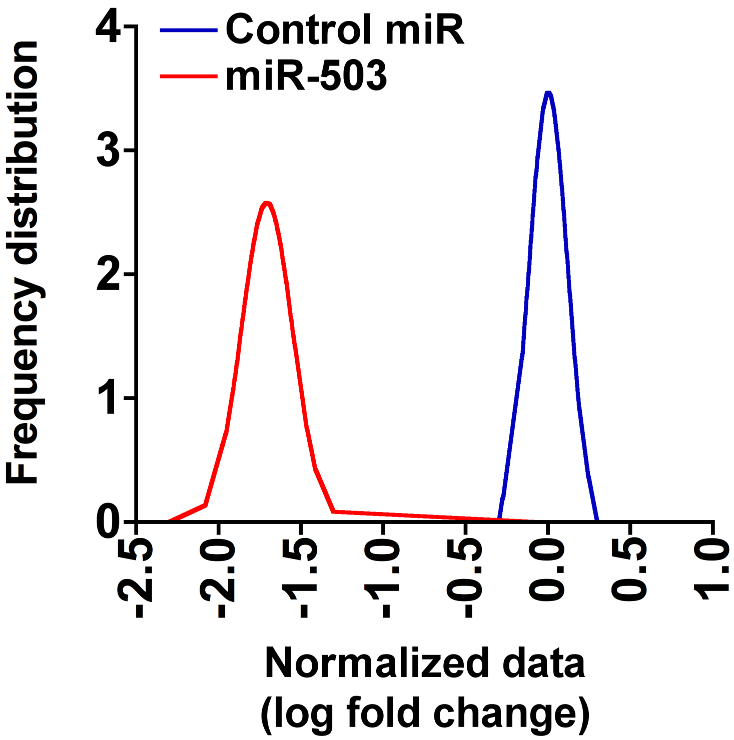

B)

Plate	*Replicate*	*Z' factor*
1	1	0.5
1	2	0.3
2	1	0.4
2	2	0.7
3	1	0.6
3	2	0.4
4	1	0.3
4	2	0.3

Figure S1. Control performance and plate QC. **A)** Graph showing the separation between the distributions of the positive (miR-503 mimic) and negative controls (miR control mimic) in the primary screen. **B)** Plate performance in the primary phenotypic screen was assessed by standard Z' factor was calculated for each screen plate using the positive and negative controls integrated into the same plate. All plates were passed (Z'factor≥0.3).

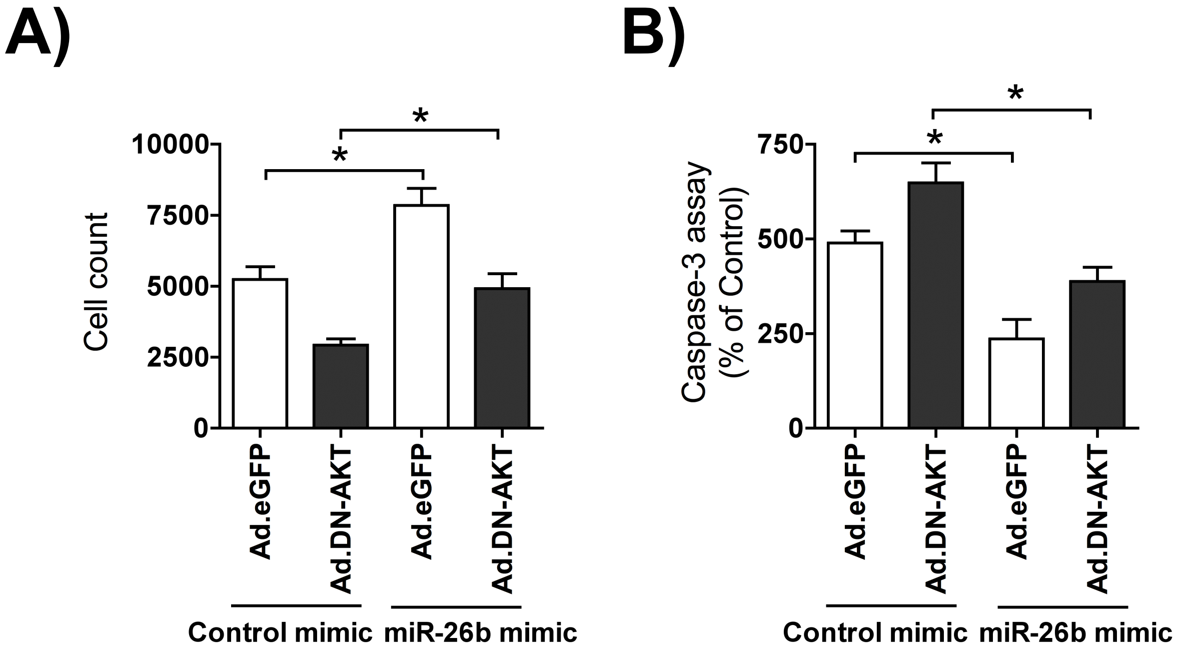

Figure S2. The proliferative and survival effect of miR-26b is mediated by AKT signaling. Effect of miR-26b mimics on cell proliferation assessed by cell count **(A)** and on apoptosis measured by caspase-3 activity **(B)** with adenovirus mediated transfer of dominant negative inhibitors of AKT (Ad.DN.AKT) or with GFP vector control (Ad.eGFP). Cells were analyzed 72 hours following miRNA mimic transfection. Error bars, mean±s.e.m., *P<0.05, (n=4 replicates derived from 3 independent experiments, unpaired t-test)

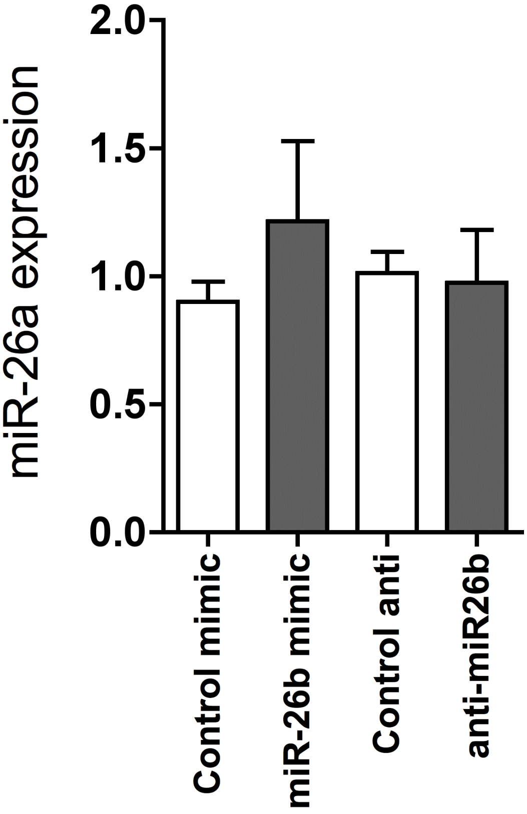

Figure S3. Effect of miR-26b overexpression and inhibition on endogenous miR-26a expression in HUVEC. RNA was isolated for performing RT-qPCR after 72 hours of transfection. miR-26b levels were normalized against snRU6 control. Error bars, mean±SEM (n=6 replicates).

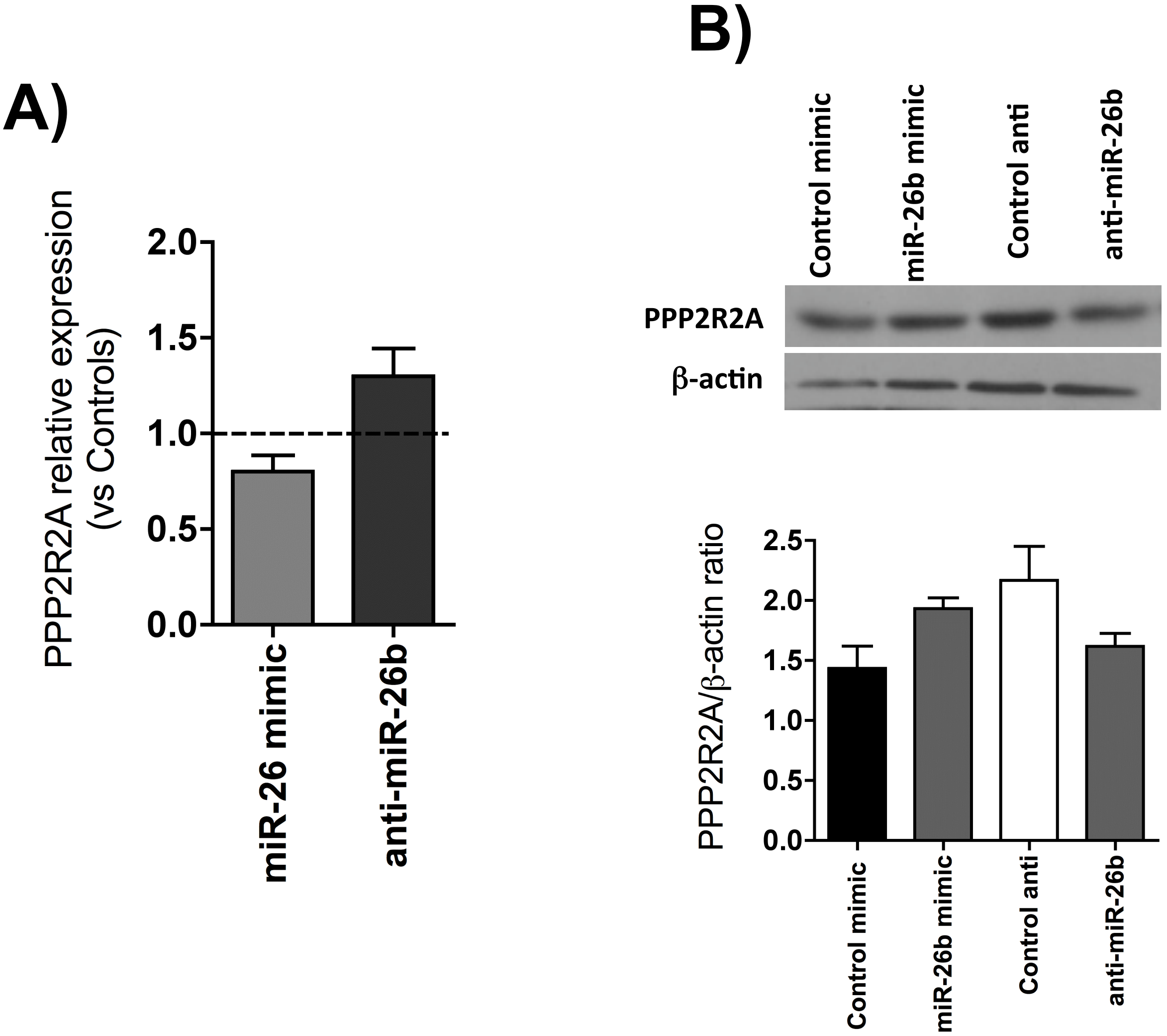

Figure S4. Validation assays for the PPP2R2A hit target of miR-26b A) HUVEC were transfected with miR-26b mimic, anti-miR-26b, miR-control mimic or anti-miR control. Three days post transfection, RNA was extracted and the levels of PPP2R2A was determined by qRT-PCR. Values were normalized to S18 and then to the controls (mimic or anti-miR). Data are shown as the mean ± SEM of 4 replicates. **B)** Transfected HUVEC with different vectors were lysed and the protein expression of PPP2R2A was analyzed by immunoblotting. β-actin was detected as a loading control. *Lower Panel*: graph represents the quantitative data of Error bars, mean±s.e.m. (n=3 replicates).

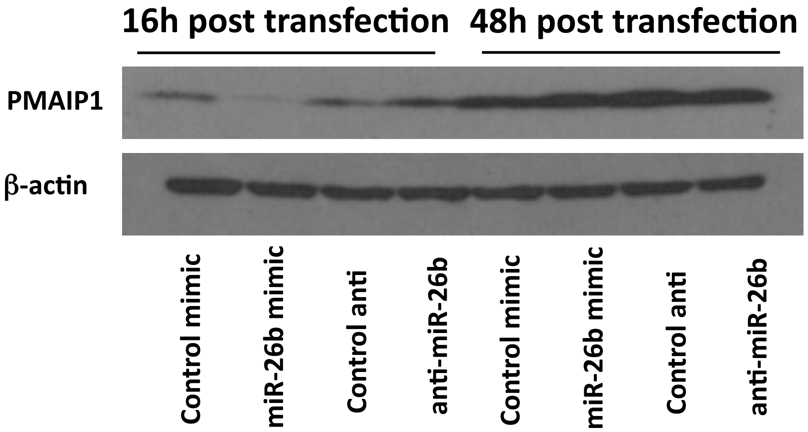

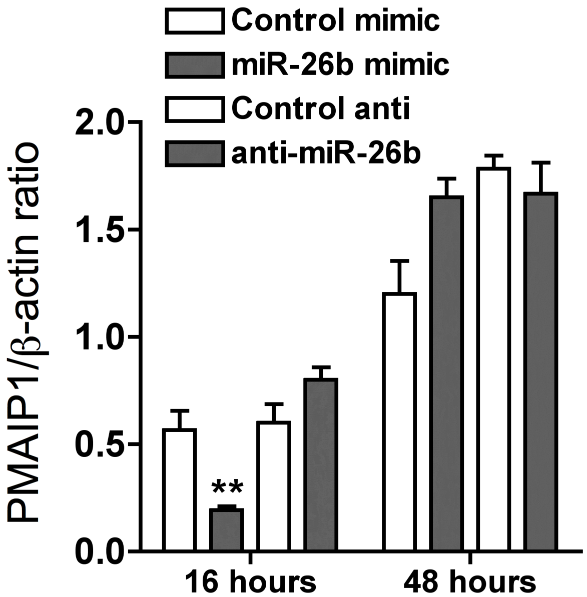

Figure S5. Time course of PMAIP1 protein expression following miR-26b overexpression or inhibition. HUVEC were transfected with miR-26b mimic, anti-miR-26b, miR-control mimic or anti-miR control. 16 hours or 28 hours post transfection, HUVEC were lysed and the protein expression of PMAIP1 was analyzed by immunoblotting. β-actin was detected as a loading control. Lower Panel: graph represents the quantitative data of. Error bars, mean±s.e.m., (**P<0.01, (n=3 replicates, unpaired t-test)

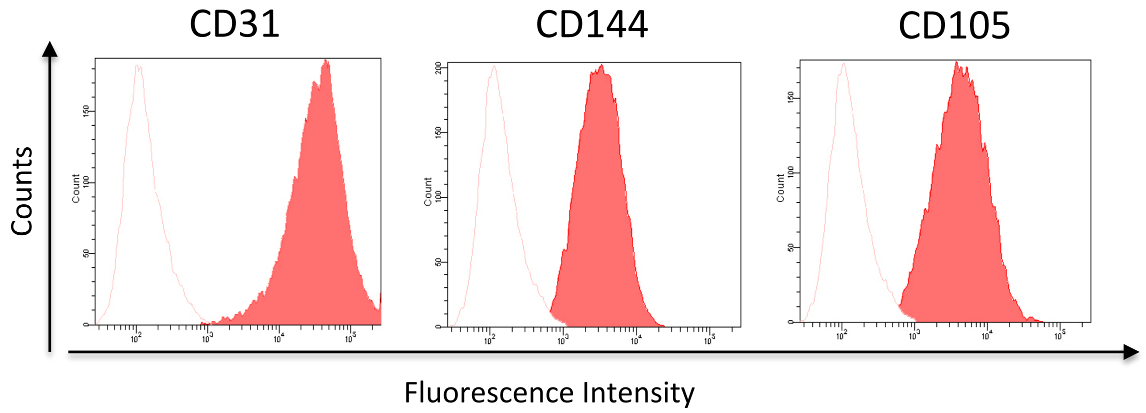

Figure S6. The expression profile of specific endothelium markers on endothelial cells (CD31^{pos}/CD45^{neg}) sorted cells from the adductor muscles using MACS® microbead system, were analyzed by Flow Cytometry. *Empty histograms* represents purified endothelial cells without primary antibody; *Filled histograms* represent the purified endothelial cells incubated with anti-CD31 (pecam1), anti-CD144 (ve-cadherin) and anti-CD105 (endoglin) antibodies.

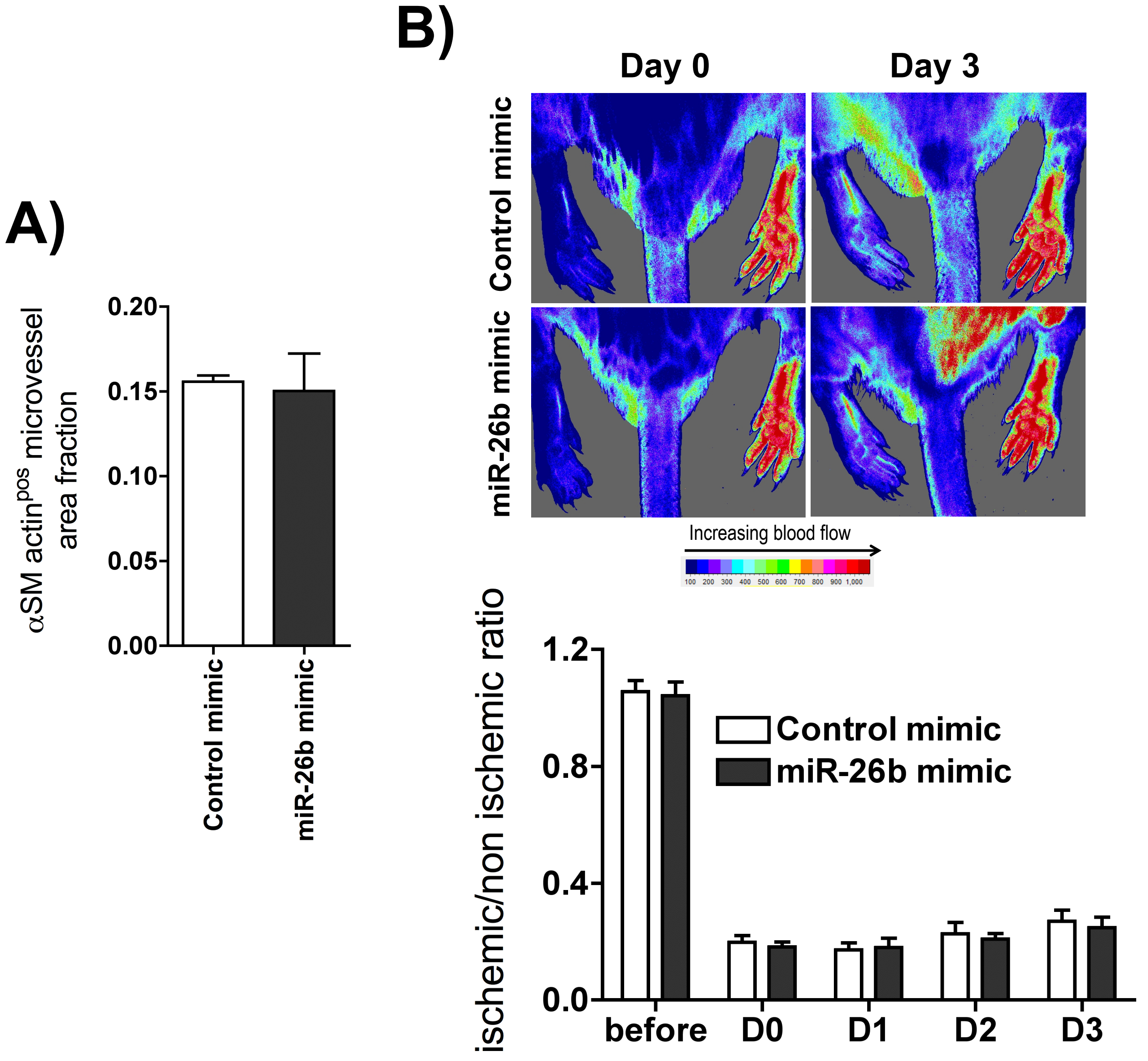

Figure S7. Effect of local miR-26b overexpression in the adductor muscle on the distal blood flow recovery in the paws and on arteriogenesis. Limb ischemia was induced by left femoral artery excision. At same occasion, miR-26b mimic or control mimic was locally injected in the adductor muscle. **A)** The area of microvessels stained with α-SM (smooth muscle) actin, which are equal or larger than 50μm were measured and normalized to the total adductor muscle area. The quantification was carried on cross-sections of ischemic adductor muscles treated with control mimic or miR-26b mimic at day 3 after ischemia injury. Data are presented as mean±SEM. (n=4 per group). **B)** Laser speckle contrast imaging was applied to measure the tissue perfusion in paws of contralateral non-ischemic limb and ischemic limb. *Lower panel*; Quantitative analysis of laser speckle contrast perfusion imaging before, immediately after the surgery and at the 1, 2, and 3 day time point presented as perfusion ratios (ischemic/non-ischemic side) in the paws. Data are presented as mean±SEM. (n=5 per group).

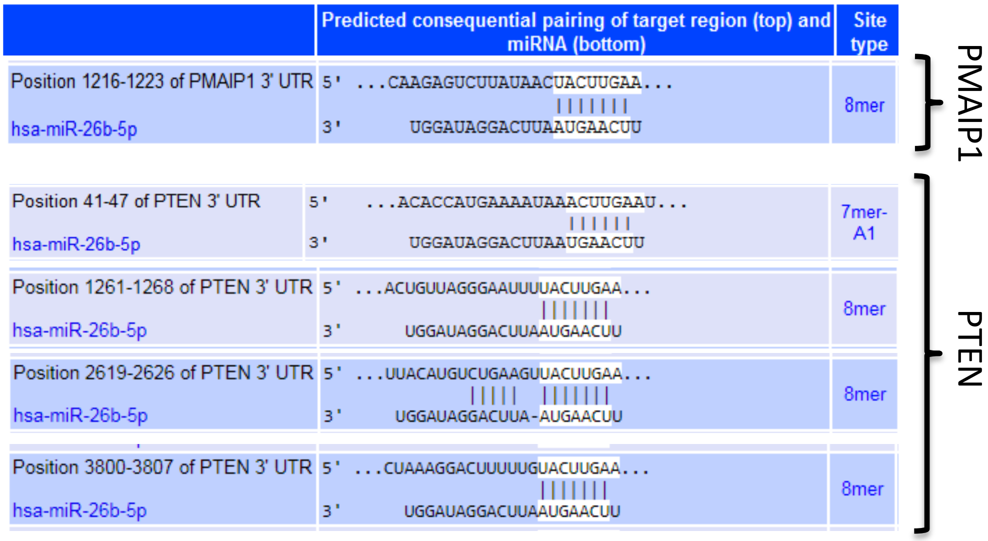

<table>
<tr><th></th><th>Predicted consequential pairing of target region (top) and miRNA (bottom)</th><th>Site type</th></tr>
<tr><td>Position 1216-1223 of PMAIP1 3' UTR
hsa-miR-26b-5p</td><td>5' ...CAAGAGUCUUAUAACUACUUGAA...
|||||||
3' UGGAUAGGACUUAAUGAACUU</td><td>8mer</td></tr>
<tr><td>Position 41-47 of PTEN 3' UTR
hsa-miR-26b-5p</td><td>5' ...ACACCAUGAAAAUAAACUUGAAU...
||||||
3' UGGAUAGGACUUAAUGAACUU</td><td>7mer-A1</td></tr>
<tr><td>Position 1261-1268 of PTEN 3' UTR
hsa-miR-26b-5p</td><td>5' ...ACUGUUAGGGAAUUUUACUUGAA...
|||||||
3' UGGAUAGGACUUAAUGAACUU</td><td>8mer</td></tr>
<tr><td>Position 2619-2626 of PTEN 3' UTR
hsa-miR-26b-5p</td><td>5' ...UUACAUGUCUGAAGUUACUUGAA...
||||| |||||||
3' UGGAUAGGACUUA-AUGAACUU</td><td>8mer</td></tr>
<tr><td>Position 3800-3807 of PTEN 3' UTR
hsa-miR-26b-5p</td><td>5' ...CUAAAGGACUUUUUGUACUUGAA...
|||||||
3' UGGAUAGGACUUAAUGAACUU</td><td>8mer</td></tr>
</table>

Figure S8. The position and sequence of miRN-26b binding sites on the PMAIP1 3'UTR and PTEN 3'UTR (extracted from TargetScan)
